# Cell-Type-Specific Dynamics of Calcium Activity in Cortical Circuits over the Course of Slow-Wave Sleep and Rapid Eye Movement Sleep

**DOI:** 10.1523/JNEUROSCI.1957-20.2021

**Published:** 2021-05-12

**Authors:** Niels Niethard, Svenja Brodt, Jan Born

**Affiliations:** ^1^Institute of Medical Psychology and Behavioral Neurobiology, University of Tübingen, Tübingen 72076, Germany; ^2^Center for Integrative Neuroscience, University of Tübingen, Tübingen 72076, Germany; ^3^German Center for Diabetes Research (DZD), Institute for Diabetes Research and Metabolic Diseases, of the Helmholtz Center Munich at the University Tübingen (IDM), Tübingen 72076, Germany

**Keywords:** calcium imaging, REM, sleep, slow oscillation, slow-wave sleep, spindle

## Abstract

Sleep shapes cortical network activity, fostering global homeostatic downregulation of excitability while maintaining or even upregulating excitability in selected networks in a manner that supports memory consolidation. Here, we used two-photon calcium imaging of cortical layer 2/3 neurons in sleeping male mice to examine how these seemingly opposing dynamics are balanced in cortical networks. During slow-wave sleep (SWS) episodes, mean calcium activity of excitatory pyramidal (Pyr) cells decreased. Simultaneously, however, variance in Pyr population calcium activity increased, contradicting the notion of a homogenous downregulation of network activity. Indeed, we identified a subpopulation of Pyr cells distinctly upregulating calcium activity during SWS, which were highly active during sleep spindles known to support mnemonic processing. Rapid eye movement (REM) episodes following SWS were associated with a general downregulation of Pyr cells, including the subpopulation of Pyr cells active during spindles, which persisted into following stages of sleep and wakefulness. Parvalbumin-positive inhibitory interneurons (PV-In) showed an increase in calcium activity during SWS episodes, while activity remained unchanged during REM sleep episodes. This supports the view that downregulation of Pyr calcium activity during SWS results from increased somatic inhibition via PV-In, whereas downregulation during REM sleep is achieved independently of such inhibitory activity. Overall, our findings show that SWS enables upregulation of select cortical circuits (likely those which were involved in mnemonic processing) through a spindle-related process, whereas REM sleep mediates general downregulation, possibly through synaptic re-normalization.

**SIGNIFICANCE STATEMENT** Sleep is thought to globally downregulate cortical excitability and, concurrently, to upregulate synaptic connections in neuron ensembles with newly encoded memory, with upregulation representing a function of sleep spindles. Using *in vivo* two-photon calcium imaging in combination with surface EEG recordings, we classified cells based on their calcium activity during sleep spindles. Spindle-active pyramidal (Pyr) cells persistently increased calcium activity during slow-wave sleep (SWS) episodes while spindle-inactive cells decreased calcium activity. Subsequent rapid eye movement (REM) sleep episodes profoundly reduced calcium activity in both cell clusters. Results indicate that SWS allows for a spindle-related differential upregulation of ensembles whereas REM sleep functions to globally downregulate networks.

## Introduction

In order to efficiently adapt to environmental conditions, the brain needs to accomplish two seemingly opposing tasks. On the one hand, it needs to encode and store relevant information in a long-lasting and stable way. This is assumed to be achieved by the strengthening of synaptic connections among the neurons representing the encoded information, thus increasing excitability of respective neuron ensembles. On the other hand, persisting encoding and storing of new information would saturate the network's limited capacities of encoding and storing which makes necessary processes that globally downregulate activity levels and synaptic connections in a homeostatic manner (Tononi and Cirelli, 2014, 2020). Sleep has been identified as a brain state that might support these two functions in an integrated manner (Klinzing et al., 2019), although the underlying mechanisms are far from being clear. As to synaptic connections, there is ample evidence supporting that sleep serves a global homeostatic process in cortical networks that down-scales and renormalizes synapses that have been strengthened during prior wake phases (de Vivo et al., 2017; Diering et al., 2017; Spano et al., 2019). Both core sleep stages, slow-wave sleep (SWS) and rapid eye movement (REM) sleep possibly contribute to this downregulation. Initially, it was thought to be particularly promoted by the <1-Hz slow oscillation (SO) as neuronal substrate underlying EEG slow-wave activity (SWA) that hallmarks SWS (Huber et al., 2004). However, based on multiunit firing activity recent findings (Grosmark et al., 2012) suggest that global down-scaling could also be conveyed by the 5- to 10-Hz theta rhythm that originates from septal-hippocampal circuits (Buzsáki, 2002) and is a hallmark of REM sleep. Beyond downregulating cortical activity, sleep is known to promote long-term memory formation and underlying strengthening of synaptic connections (Yang et al., 2014; Sawangjit et al., 2018; Klinzing et al., 2019; Niethard and Born, 2019). Again, both core sleep stages, SWS and REM sleep appear to be implicated in this process. Enhancing SOs during SWS enhances long-term memory formation (Chauvette et al., 2012; Ngo et al., 2013; Kim et al., 2019), although this effect could primarily reflect the driving force of SOs on thalamic spindles (Latchoumane et al., 2017). Spindles are waxing and waning oscillations in the 10–15 Hz range that often nest into the upstate of the SO (Puentes-Mestril et al., 2019; Fernandez and Lüthi, 2020; Oyanedel et al., 2020), and appear to provide optimal conditions for synaptic plastic processes underlying memory formation in cortical microcircuits (Rosanova and Ulrich, 2005; Seibt et al., 2017; Niethard et al., 2018). Finally, there are also studies indicating that memory formation profits from REM sleep and accompanying theta activity (Rasch and Born, 2013; Boyce et al., 2016; Niethard et al., 2017).

Although SWS and REM sleep have been connected to both processes of global downregulation of cortical activity as well as to the selective upregulation of activity and synapses underlying memory formation, little is known about how these sleep stages and associated oscillatory phenomena concurrently establish the two functions in cortical networks. Here, we used *in vivo* two-photon calcium imaging in mice to assess the temporal calcium activity dynamics in large populations of cortical layer 2/3 cells across episodes of SWS and REM sleep, with the aim to dissociate neuronal subpopulations whose activity is down-scaled or up-scaled during sleep. Two-photon calcium imaging additionally allowed us to differentiate calcium activity changes in the major population of excitatory cells, i.e., pyramidal (Pyr) cells, and the two major populations of inhibitory interneurons, i.e., parvalbumin-positive interneurons (PV-In) and somatostatin-positive interneurons (SOM-In). We found that Pyr cells representing ∼80% of cortical neurons, indeed significantly decreased calcium activity in the course of SWS and REM sleep episodes. The decrease during SWS, but not during REM sleep, was accompanied by an increase in calcium activity of PV-In. Importantly, contrasting with the general downregulation of Pyr calcium activity during SWS, a subpopulation of these cells showing highest calcium activity during spindles, upregulated activity in the course of SWS. Surprisingly, these spindle active cells possibly involved in memory formation, also underwent profound downregulation during succeeding REM sleep.

## Materials and Methods

### 

#### Animals

For the experiments two different strains of transgenic mice, PV-Cre mice (RRID:IMSR_JAX:008069; *n* = 4) and SOM-Cre mice (RRID:IMSR_JAX:013044; *n* = 4) were used. All mice were housed in groups of up to five animals under temperature-controlled and humidity-controlled conditions (22 ± 2°C; 45–65%) and a 12/12 h light/dark cycle. All recordings started during the first hour of the light phase and only male mice older than eight weeks were recorded. Procedures were the same as described previously (Niethard et al., 2016, 2018), and also datasets were from the same mice used in these previous studies. All experiments were approved by the local institutions in charge of animal welfare (Regierungspräsidium Tübingen, State of Baden-Wuerttemberg, Germany).

#### Surgery

All animals were anesthetized with 0.1 mg/g ketamine and 0.008 mg/g xylazine with a supplement of isoflurane. For topical anesthesia lidocain was applied. Afterwards the animals were mounted on a stereotaxic frame. Body temperature was continuously monitored and maintained at 37°C. A custom-made headpost was glued to the skull and subsequently cemented with dental acrylic (Kulzer Palapress).

Virus injection and the implantation of the imaging window followed headpost implantation. To this end, a craniotomy above the sensorimotor cortex (1.1 mm caudal and 1–1.3 mm lateral from the bregma) with a size of 1.2 × 2 mm was made. Afterwards, two viruses (AAV2/1-syn-GCaMP6f 2.96 × 10^12^ genomes/ml and AAV2/1-Flex-tdTomato 1.48 × 10^11^ genomes/ml) were injected into multiple sites of the area of craniotomy (10–20 nl/site; 3–5 min/injection). The injection depth was between 130 and 300 µm. Virus injection was followed by the implantation of the imaging window (1 × 1.5 mm). The space between the skull and the imaging window was filled with agarose (1.5–2%), and then the imaging window was cemented with dental acrylic.

EEG electrodes were implanted on the cortical surface of the contralateral hemisphere relative to the imaging window (−2.5 mm, lateral +2.5 mm from bregma). The reference electrodes were implanted on the brain surface 1 mm relative to λ. Two wire electrodes were implanted into the neck muscle for EMG recordings (Science Products). After the surgery, all animals were brought back to their home cage and were single-housed for the rest of the experiments. They had at least 10 d of recovery from surgery before imaging sessions started.

#### Head fixation procedure

After handling the animals 10 min/d for one week, the animal was habituated to the head fixation. Habituation consisted of four sessions per day for one week with increasing fixation durations (30 s, 3 min, 10 min, and 30 min) interleaved by 10-min rest intervals. Habituation was conducted until 24 h before the first imaging session during the early light phase.

#### EEG and EMG recordings

Sleep stages were identified based on EEG and EMG recordings during the imaging sessions. EEG and EMG signals were amplified, filtered (EEG: 0.01–300 Hz; EMG: 30–300 Hz), and sampled at a rate of 1000 Hz (amplifier: model 15A54; Grass Technologies). Based on EEG/EMG signals for succeeding 10-s epochs, the brain state of the mouse was classified into wake, SWS, and REM sleep stages. Sleep stage classification was supported using the software SleepSign for animals (Kissei Comtech).

#### Detection of sleep SOs and spindles

For the detection of discrete SO and spindle events during SWS, algorithms were adopted from previous studies (David et al., 2013). In brief, for the detection of SOs, the EEG was bandpass filtered between 0.1 and 4.5 Hz and then, all positive-to-negative zero crossings of the signal as well as the local minimum and maximum between each two successive crossings were marked. Intervals between two succeeding positive-to-negative zero-crossings were identified as a SO event when the length of this interval was between 0.4 and 2 s and when the minimum amplitude and minimum-to-maximum amplitude was >66.6% of the average of the respective amplitude values across the whole recording. For these remaining events the enclosing zero-crossings represent the onset and end of the corresponding SO cycle.

For the detection of spindles, the EEG signal was band pass filtered between 7 and 15 Hz, rectified and, subsequently, all maxima were interpolated to yield the envelope for this frequency band. A spindle event was identified whenever the envelope exceeded an individual threshold for a duration of 0.5–3.0 s, whereby the threshold was defined by the SD of the filtered signal during all SWS episodes of an individual mouse multiplied by a factor of 1.5. Thus, the positive and subsequent negative threshold crossing represented the onset and end of a spindle event, respectively. Previous studies showed that with this criteria larger spindle events are identified that typically occur in parallel in both hemispheres (Oyanedel et al., 2020).

For calculating correlations between measures of EEG activity (power and energy) in the frequency bands of interest (SWA: 0.1–4 Hz, spindle; 7–15 Hz, theta: 4–10 Hz) and ΔF/F signals of calcium activity, the EEG signals were filtered in the respective frequency bands using a third order Butterworth filter, and the root mean square of the signal was used as power measure. The energy within the respective frequency band was calculated by integrating the root mean square of the filtered signal over the duration of the sleep episode. Changes during a sleep episode in EEG power and ΔF/F signals, respectively were calculated by subtracting the mean value during the first of the episode from that during the 3rd third of the episode.

To discriminate spindle-active and spindle-inactive cells during each SWS episode, the difference between the mean ΔF/F signals during the detected spindle events (i.e., the mean signal between spindle onset and offset) and during the remaining periods of the episode (without the detected spindle events) was calculated for each cell. This difference was separately calculated for each individual SWS episode. The top 20% cells with the largest difference between mean ΔF/F signal during spindles and the corresponding SWS episode were considered as spindle-active cells, whereas the 20% cells with the smallest difference were considered as spindle-inactive. We decided for the 20% criterion to ensure that effects did not derive from an unlabeled interneuron population expected to represent <10% of all unlabeled cells (Markram et al., 2004; Jiang et al., 2015). Although the criterion is arbitrary to a certain extent, results did not essentially change with less (e.g., 25%) or more stringent criteria (10%). SO-active and SO-inactive cells were defined in an analogous manner, using the difference between ΔF/F signals during the SO upstate (defined by a 1-s interval beginning with the SO downstate peak) and during the remaining period of the SWS episode without SOs.

#### Two-photon imaging

*In vivo* imaging was performed using a two-photon microscope based on the MOM system (Sutter) controlled by ScanImage software (Pologruto et al., 2003). The light source was a pulsed Ti:sapphire laser (λ = 980 nm; Chameleon; Coherent). Red and green fluorescence photons were collected with an objective lens [Nikon; 16×; 0.80 numerical aperture (NA)], separated by a 565 nm dichroic mirror (Chroma; 565dcxr) and barrier filters (green: ET525/70 m-2p; red: ET605/70 m-2p), and measured using photomultiplier tubes (Hamamatsu Photonics; H10770PA-40). Imaging frames were visually inspected to exclude cross talk between green and red channels. The imaging frame consisted of 1024 × 256 pixels, and the frame rate was 5.92 Hz (169 ms per frame). Images were collected in layer 2/3 at a depth of 150–250 μm. All subsequent analyses were performed on the original data or after high-pass prefiltering at 0.1 Hz to eliminate slower changes possibly originating from metabolic and blood flow changes. As both analyses yielded essentially the same results, and also because of the lower sensitivity to changes in blood flow of two-photon imaging, this report is restricted to analyses based on unfiltered two-photon imaging data.

#### Two-photon image analysis

Lateral motion was corrected in two steps. A cross-correlation-based image alignment (Turboreg) was performed, followed by a line-by-line correction using an algorithm based on a hidden Markov model (Dombeck et al., 2007). Regions of interest (ROIs) containing individual neurons were drawn manually, and the pixel values within each ROI were summed to estimate the fluorescence of this neuron. PV-INs and SOM-INs were manually detected by red fluorescence signal expressed by AAV2/1-Flex-tdtomato. The individual cell traces were calculated as the average pixel intensity within the ROIs for each frame. The cell traces were transformed into the percent signal change (ΔF/F), in which the baseline for each cell was defined as the 20th percentile value of all frames. Importantly, rather than extracting calcium spikes, we decided to base our analyses on the original fluorescence signal which represents a standard procedure with the advantage that it also covers subthreshold calcium activity of neurons (Cox et al., 2016; Niethard et al., 2016). To confirm that the neuropil signal did not affect our results and to compensate for background noise we performed a standard neuropil subtraction for each cell's fluorescence trace (Cox et al., 2016; Niethard et al., 2018). The neuropil signal was estimated for each ROI as the average pixel value within two pixels around the ROI (excluding adjacent cells). The true signal was estimated as F(t) = FinROI − r × FaroundROI, where *r* = 0.7.

#### Immunohistochemistry

After finishing the experiments mice were deeply anesthetized (0.3 mg/g ketamine and 0.024 mg/g xylazine, i.p.) and with 4% paraformaldehyde in 0.1 m PBS (4% PFA) intracardially perfused. Then the brains were postfixed in 4% PFA at 4°C overnight, and rinsed 3 × with 0.1 m PBS. Coronal slices (thickness 65 μm) were blocked in 10% normal goat serum (NGS; Jackson ImmnunoResearch) and 0.3% Triton X-100 (Sigma-Aldrich) in 0.1 m PBS for 1.5 h at room temperature. Slices were incubated with anti-PV rabbit primary antibody (1:1000; #24428, Immunostar, RRID: AB_572259) or anti-SOM rabbit primary antibody (1:1000; #T-4547, Peninsula Laboratories, RRID: AB_518618) in carrier solution (2% NGS and 0.3% Triton X-100 in PBS) for 48 h at 4°C. Following 4 × 10 min rinses with 0.1 m PBS the slices were incubated in goat anti-rabbit IgG antibodies conjugated either with Alexa Fluor 405 (for PV-In staining, AB_221605) or Alexa Fluor 633 (for SOM-In staining, AB2535732; both from Thermo Fisher Scientific, 1:1000) in carrier solution for 3 h at room temperature on the shaker. Images were acquired on a confocal microscope (LSM 710, Carl Zeiss). Overall, the fraction of cells only expressing Alexa Fluor but not tdtomato and GCamp6f for PV-In and SOM-In was each below 2%.

#### Statistics

All analyses referred to differences within episodes of a specific brain state (SWS, REM, wake episodes) or between neighboring episodes. ΔF/F signals of each detected cell were normalized by dividing the signal value by the cell's mean calcium activity during all episodes scored as wakefulness. (Only cell clustering and comparisons of calcium activity during wake episodes themselves was based on non-normalized ΔF/F signals.) Based on previous work that, like the present study, aimed at characterizing slow trends across the entire duration of SWS and REM sleep episodes (Grosmark et al., 2012), we divided each episode of SWS, REM sleep, and wakefulness into thirds, and averaged calcium activity for each cell during the 1st, 2nd, and 3rd third of each episode. For analyzing the temporal calcium activity dynamics, ANOVAs were performed including the repeated measures factor “1st/3rd third” (representing calcium activity during the first and last third of an episode), and the group factors “SWS/REM” (representing the different sleep stages) and “active/inactive” (representing the cell clusters formed on the basis of different calcium activity levels). ANOVAs run on neighboring SWS episodes contained a repeated measures factor SWS-1/SWS-2. Significant ANOVA effects were followed by *post hoc* (two-sided) pairwise comparisons using Student's *t* test and, to account for non-normal distributions, Wilcoxon's sign-rank test. Since both tests revealed essentially the same results, here, we only report results from Wilcoxon's tests. To account for the hierarchical structure of the data, as recorded cells are clustered within animals, we additionally performed mixed effect modeling with random effects for animals, in R using the lme4 package. Model assumptions, i.e., linearity, homogeneity of variance and homoscedasticity were confirmed, and model testing was done by approximating the likelihood ratio test to compare models with and without interaction term. These hierarchical analyses confirmed essentially all ANOVA results reported here and, hence, will not be separately reported. For correlation analyses, Pearson product-moment correlation coefficients were calculated. Respective *p* values were Bonferroni corrected.

## Results

### Sleep reduces calcium activity of Pyr cells and simultaneously widens the activity distribution

We used *in vivo* two-photon calcium imaging of layer 2/3 soma of neurons within sensorimotor cortex in PV-Cre or SOM-Cre transgenic mice. To discriminate calcium activity of PV-In and SOM-In, respectively, we injected two different virus types. First, we injected GCaMP6f to express a genetically encoded calcium indicator. Second, for discriminating the interneurons from other cells, we injected a virus expressing tdTomato Cre-dependently (AAV-FLEX-tdTomato). After 10–14 d, almost all neurons around the injection site (∼200 μm) expressed GCaMP6f, whereas tdTomato expression was selective for the specific interneuron type. The majority (∼80%) of unlabeled cells in this setup consists of Pyr cells; we, therefore, considered this population as putative Pyr cells. Once the GCaMP6f expression was strong enough for sufficient image quality, the animals were habituated to head fixation and then repeatedly recorded during episodes of wakefulness, SWS, and REM sleep. Extended Data Figs. 1-1, 1-2 summarize the mean duration of sleep stage episodes imaged and the total imaging time per session, respectively.

**Figure 1.**
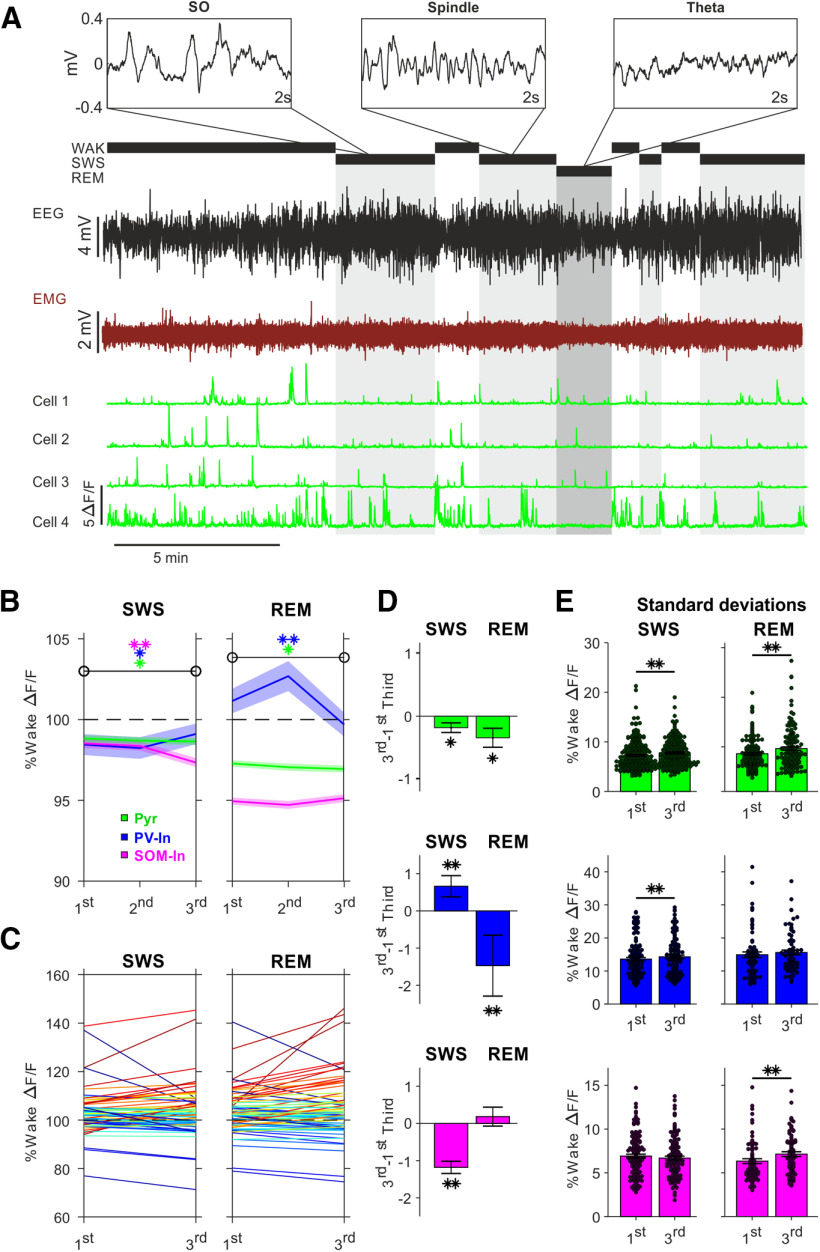
Cortical calcium activity decreases and disperses during sleep. ***A***, Example recording period containing wake, SWS, and REM sleep episodes, with associated EEG (black) and EMG recordings (red). Top boxes show with magnified resolution example EEG traces of SOs, spindles and REM theta. Bottom traces, Corresponding ΔF/F calcium traces from four Pyr cells. ***B***, Mean ± SEM wake-normalized ΔF/F calcium signals of Pyr (green), PV-In (blue), and SOM-In (pink) during thirds of SWS (*n*: Pyr 255, PV-In 132, SOM-In 123; in total 560.37 min) and REM sleep episodes (*n*: Pyr 145, PV-In 71, SOM-In 74; in total 196.85 min). Significant changes from 1st to last third of SWS and REM episodes are indicated by asterisks in the respective color (**p < 0.01, *p < 0.05; for distribution of changes, see Extended Data Figure 1-1). ***C***, Wake normalized ΔF/F calcium signals of Pyr cells during the first (1st) and last (3rd) third of an example SWS and subsequent REM episode. Increasing or decreasing courses for each recorded cell are color coded from red to blue, respectively. ***D***, Mean difference (±SEM) in calcium activity of Pyr, PV-In, and SOM-In from 1st to last third (3rd–1st third) of SWS (left) and REM sleep (right) episodes (**p < 0.01, *p < 0.05). ***E***, Mean ± SEM of SDs of calcium activity during 1st and last third of SWS (left) and REM sleep episodes (right) for Pyr (green), PV-In (blue), and SOM-In (**p < 0.01, *p < 0.05). In all panels, the wake-normalized ΔF/F calcium signal is indicated (in %) with the signal across all wake episodes set to 100%. Data were recorded from four PV-cre and four SOM-cre animals (for details, see Extended Data Figures 1-1, 1-3).

10.1523/JNEUROSCI.1957-20.2021.f1-1 Extended Data Figure 1-1 Distributions of changes in calcium activity during episodes of SWS and REM sleep. Distributions of changes in the wake normalized ΔF/F calcium signals from 1st to last third (3rd–1st third) of SWS (left) and REM sleep (right) episodes for Pyr (green), PV-In (blue), and SOM-In (pink); *y*-axis indicates number of cells, *x*-axis change in normalized ΔF/F signal. Download Figure 1-1, TIF file.

**Figure 2.**
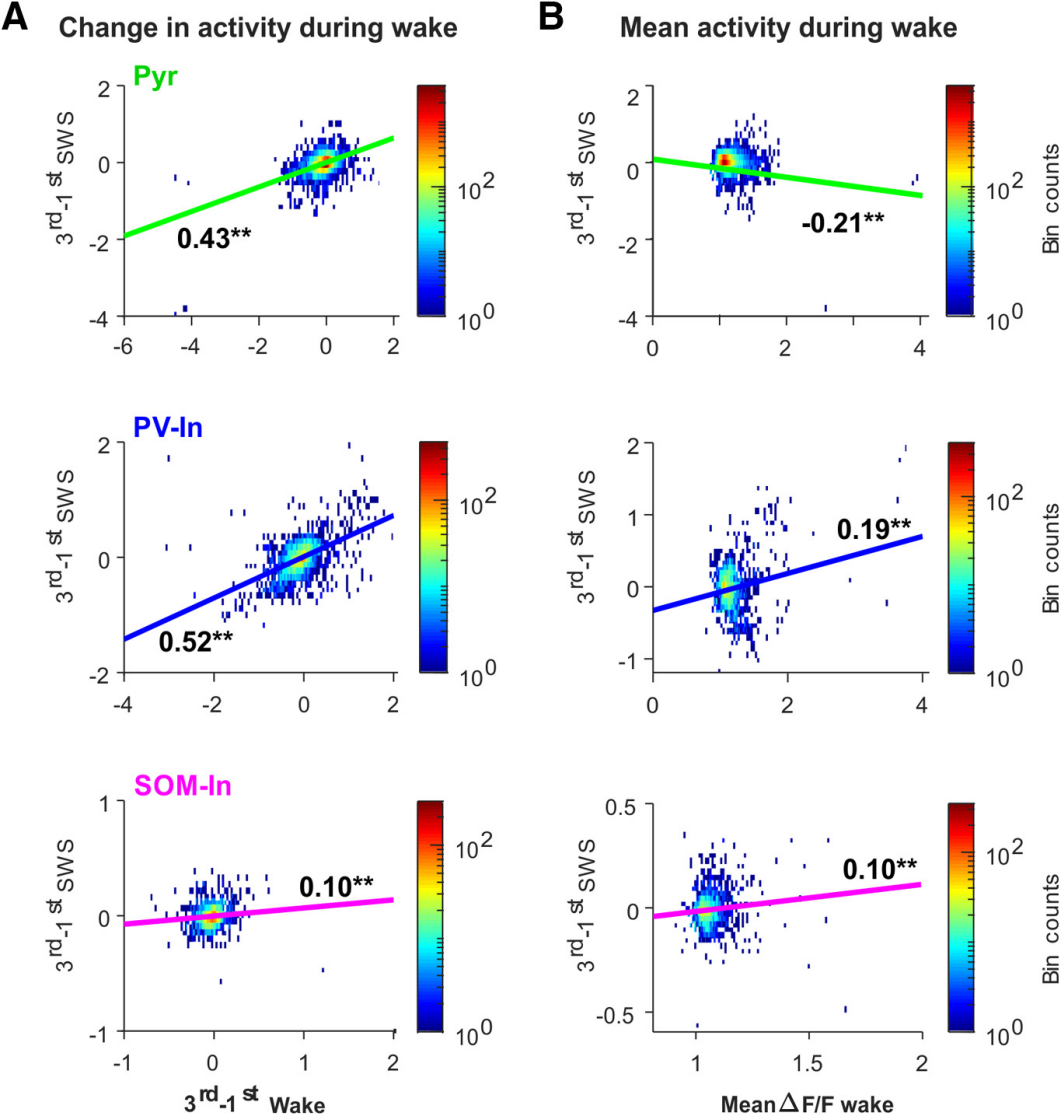
Changes in the cell's calcium activity during SWS correlate with changes in calcium activity during prior wake episode. Density plots for correlations between the change in a cell's calcium activity during a SWS episode (3rd–1st third) and (***A***) the change in calcium activity during the preceding wake episode (3rd–1st third), and (***B***) the mean calcium activity level during the preceding wake episode for (from top to bottom) Pyr, PV-In, and SOM-In; *r* values and significances are indicated (**p < 0.01, *p < 0.05). Note, increases in Pyr and PV-In during SWS episodes show robust correlations with increases in calcium activity of these cells during the prior wake episodes.

10.1523/JNEUROSCI.1957-20.2021.f1-2 Extended Data Figure 1-2 Mean ± SEM SWS and REM sleep episode duration for PV-cre and SOM-cre animals in seconds. Note, sleep stage episodes <30 s were excluded from analyses. Download Figure 1-2, DOCX file.

**Figure 3.**
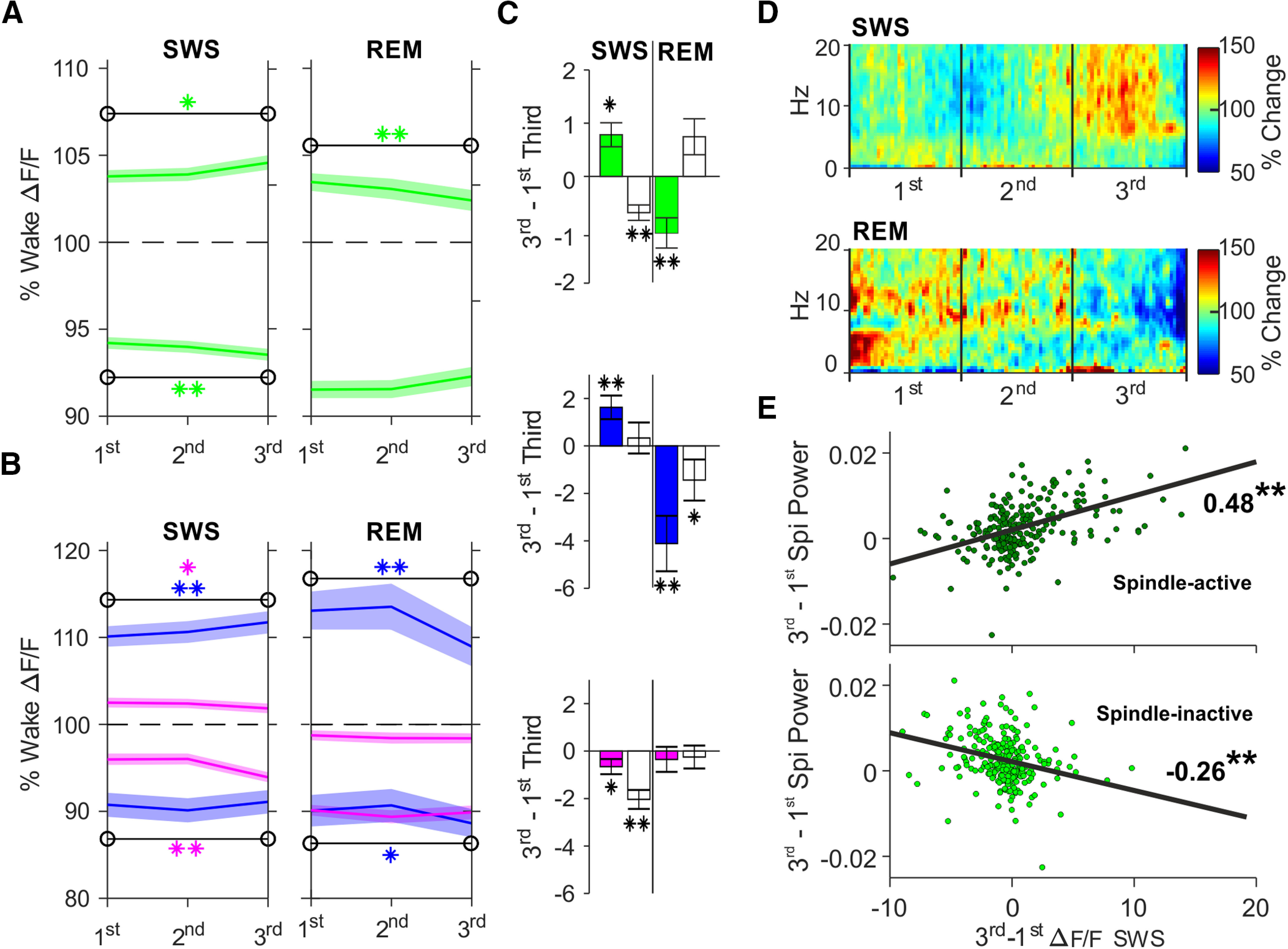
Spindle-active Pyr cells show increased calcium activity during SWS. ***A***, Mean ± SEM wake-normalized ΔF/F calcium signals of spindle-active (upper traces) and spindle-inactive (lower traces) Pyr cells (green) during thirds of SWS (*n*: Pyr 255, PV-In 132, SOM-In 123) and REM sleep episodes (*n*: Pyr 145, PV-In 71, SOM-In 74). Significant changes from 1st to last third of SWS and REM episodes are indicated by asterisks (**p < 0.01, *p < 0.05). ***B***, As in ***A***, but for spindle-active PV-In (upper lines, blue) and SOM-In (pink) and spindle-inactive PV-In and SOM-In (lower blue and pink lines, respectively). ***C***, Mean difference (±SEM) in calcium activity of spindle active (filled bars) and spindle-inactive (empty bars) Pyr, PV-In, and SOM-In from 1st to last third (3rd–1st third) of SWS (left) and REM sleep (right) episodes (**p < 0.01, *p < 0.05). Note, spindle-active Pyr and PV-In significantly increase calcium activity over SWS episodes and significant decrease over REM sleep episodes whereas wake-active Pyr cells decrease calcium activity during SWS episodes (Extended Data Figure 3-1). ***D***, Time-frequency plots of EEG power (0.1–20 Hz) across all SWS (top) and REM sleep (bottom) episodes. Color code indicates change (in %) in power with reference to the mean power during an episode in the respective frequency. ***E***, Density plots for correlations between calcium activity changes during SWS (3rd–1st third) and changes in the spindle band (7–15 Hz) power for spindle-active (top) and spindle-inactive (bottom); *r* values and significances are indicated (**p < 0.01).

10.1523/JNEUROSCI.1957-20.2021.f1-3 Extended Data Figure 1-3 Total imaging time per session for PV-cre and SOM-cre animals in minutes. The total imaging time was for PV-cre animals 31.75 h, and for SOM-cre animals 40.26 h. Download Figure 1-3, DOCX file.

We characterized the calcium activity dynamics of the neuron populations of interest (Pyr, PV-In, SOM-In) during episodes of SWS and REM sleep, by comparing activity during the first and last third of the individual episodes (Fig. 1). In these analyses, the mean calcium activity of an individual cell during all wake episodes (set to 100%) was used to normalize calcium activity. Pyr cells showed a decrease in calcium activity during both, SWS and REM sleep, from the first to the last third of an episode (*p* < 0.05; SWS: *z* = −2.22; REM: *z* = −2.45). PV-In cells increased their calcium activity in the course of SWS episodes (*p* < 0.001; *z* = 2.976), but significantly decreased calcium activity from the first to the last third of REM sleep episodes (*p* < 0.01; *z* = −2.65). SOM-In cells showed, opposite to PV-In, significantly decreasing calcium activity during SWS (*p* < 0.001; *z* = −6.2) whereas during REM episodes, calcium activity levels remained unchanged (*p* > 0.85; Extended Data Fig. 1-1). As previously reported (Niethard et al., 2016), calcium activity of Pyr and SOM-In cells was generally higher during SWS than REM sleep, whereas PV-In calcium activity was distinctly higher during REM sleep than SWS.

Changes in calcium activity during SWS neither correlated with SWS episode duration (*p* > 0.8) nor with SWA energy (i.e., power integrated over time) or changes in SWA during these episodes for any of the three cell types (*p* > 0.11). Decreases in Pyr calcium activity during REM sleep were the stronger the higher EEG theta energy was during the REM episode (*r* < −0.25, *p* < 0.01), and the same held for decreases in PV-In calcium activity over REM episodes (*r* < −0.37, *p* < 0.01). Moreover, decreases in Pyr and PV-In calcium activity were the more pronounced the longer the duration was of a REM episode (Pyr: *r* = −0.35, *p* < 0.001; PV-In: *r* = −0.43, *p* < 0.001).

That Pyr cells, i.e., the major population of cortical neurons, on average, decrease in calcium activity over SWS and REM sleep episodes is consistent with the view that sleep globally downregulates cortical activity (Niethard et al., 2017; Tononi and Cirelli, 2020). However, given that sleep serves twofold functions, to homeostatically downregulate circuit activity but enhance circuits involved memory formation, we hypothesized, that sleep would not equally affect all cells. To test this hypothesis, in a first approach, we compared the SD of the ΔF/F calcium signal level for the different cell types between the first and last third of SWS and REM sleep episodes. Contrary to an expected statistical regression to the mean, we found that the variability in calcium activity of the Pyr cell population, indeed, robustly increased from the first to the last third of both, SWS and REM sleep episodes (*p* < 0.01; SWS: *z* = 4.83; REM: *z* = 3.86, Fig. 1*C*). PV-In populations showed increases in variability of the ΔF/F calcium signal level only during SWS episodes (*p* < 0.001; *z* = 3.45), and SOM-In only during REM episodes (*p* < 0.001; *z* = 2.97).

### Does prior wake activity predict the cell's activity during subsequent sleep?

Which neurons do and which do not downregulate activity during sleep? To answer this question, in a first analysis we correlated calcium activity dynamics during wake episodes with that during succeeding sleep episodes, assuming that cells strongly increasing their calcium activity over a wake episode would be implicated in information encoding. We found, particularly for Pyr and PV-In, positive correlations between the change in calcium activity during a wake episode (ΔF/F calcium signal level during last third minus level during 1st third of a wake episode) and the calcium activity changes during the subsequent SWS episode (Pyr: *r* = 0.43, *p* < 0.001; PV-In: *r* = 0.52, *p* < 0.001; SOM-In: *r* = 0.10, *p* < 0.001; Fig. 2*A*). Thus, cells showing pronounced increases in calcium activity over a wake episode tend to increase their activity during subsequent SWS and vice versa.

Of note, different correlations were revealed for the mean calcium activity level the cells showed during the prior wake episode. Here, Pyr cells with higher calcium activity during the wake episode displayed greater decreases during subsequent SWS (*r* = −0.21; *p* < 0.001; Fig. 2*B*). Likewise, the comparison of wake-active cells (i.e., the 20% most active cells during the wake episode preceding the respective SWS episode) with wake-inactive cells (lowest 20% of cells) revealed that wake-active Pyr diminished calcium activity in the course of SWS episodes (*p* < 0.001; *z* = −4.97), while wake-inactive Pyr did not change their calcium activity (*p* > 0.18; *F* = 28.45, *p* < 0.001, for 1st/3rd × active/inactive ANOVA interaction, Extended Data Fig. 3-1).

### Spindle-active Pyr cells increase calcium activity during SWS

Spindles are well-known to be involved in sleep-dependent memory formation and underlying cortical synaptic plasticity (Puentes-Mestril et al., 2019). We therefore hypothesized that neurons active during spindles are the ones that are spared from downregulation or even show increasing calcium activity during SWS. To discriminate spindle-active and spindle-inactive cells during each SWS episode, the difference between the mean ΔF/F signals during the detected spindle events (i.e., the mean signal between spindle onset and offset) and during the remaining periods of the episode (without the detected spindle events) was calculated for each cell. This difference was separately calculated for each individual SWS episode. The top 20% cells with the largest difference between mean ΔF/F signal during spindles and the corresponding SWS episode were considered as spindle-active cells, whereas cells with small calcium activity differences within the lowest 20% were considered spindle-inactive. We then analyzed calcium activity changes across each SWS episode. Indeed, spindle-active Pyr and also spindle-active PV-In cells increased their calcium activity level from the first to the last third of a SWS episode (*p* < 0.05; Pyr: *z* = 2.00; PV-In: *z* = 3.42), while spindle-active SOM-In decreased calcium activity within SWS episodes (*p* < 0.05, *z* = −2.01; Fig. 3). A control analysis on the overall population of Pyr cells confirmed that the majority (70%) of Pyr cells that showed a significant increase in calcium activity during a SWS episode (with significance defined here by an increase >1.96 SDs from the mean change in the population) also belonged to the identified population of “spindle-active” Pyr cells. During REM sleep episodes, spindle-active Pyr and PV-In decreased calcium activity (*p* < 0.001; Pyr: *z* = −4.15; PV-In: *t* = −3.63, df *=70*), while SOM-In changes remained non-significant.

10.1523/JNEUROSCI.1957-20.2021.f3-1 Extended Data Figure 3-1 Pyr cells showing generally high calcium activity during wakefulness reduce their activity during SWS. ***A***, Mean ± SEM wake-normalized ΔF/F calcium signals of wake-active (upper lines) and wake-inactive (lower lines) Pyr cells (green) during thirds of SWS (*n*: Pyr 255, PV-In 132, SOM-In 123) and REM sleep episodes (*n*: Pyr 145, PV-In 71, SOM-In 74). Significant changes from 1st to last third of SWS and REM episodes are indicated by asterisks in the respective color (**p < 0.01, *p < 0.05). ***B***, As in ***A***, but for wake-active PV-In (upper lines, blue) and SOM-In (pink) and wake-inactive PV-In and SOM-In (lower blue and pink lines, respectively). ***C***, Mean difference (±SEM) in calcium activity of wake-active (filled bars) and wake-inactive (empty bars) Pyr, PV-In, and SOM-In from 1st to last third (3rd–1st third) of SWS (left) and REM sleep (right) episodes (**p < 0.01, *p < 0.05). “Wake-active” cells refer to the top 20% of the cells (of the respective population) with the highest average calcium activity during the wake episode preceding the respective SWS episode. Correspondingly, “wake-inactive” cells refer to the 20% cells with the lowest calcium activity during this wake episode. Download Figure 3-1, TIF file.

Spindle-inactive Pyr displayed calcium dynamics opposite to those of spindle-active Pyr cells, i.e., they reduced calcium activity during SWS episodes (*p* < 0.001, *z* = −5.30). During REM sleep their calcium activity remained unchanged (*p* > 0.1, *z* = 1.63; *F* = 45.17, *p* < 0.001, for SWS/REM × Active/inactive ANOVA interaction). Spindle-inactive PV-In did not change calcium activity during SWS episodes (*p* > 0.4) and increased activity during REM episodes (*p* < 0.05, *z* = −2.07). Spindle-inactive SOM-In followed the same activity pattern as spindle-active SOM-In with decreasing calcium activity during SWS (*p* < 0.001, *z* = −5.23), and unchanged calcium activity during REM episodes (*F* = 5.89, *p* < 0.05, for SWS/REM ANOVA main effect). Supplementary analyses revealed that the observed dynamics remained the same when spindle-active and inactive cells were separately clustered according to whether or not the spindle occurred in the presence of a SO. Moreover, we found for all three cell types that, compared with spindle-inactive cells, the spindle-active cells also showed increased calcium activity during the immediately preceding wake episode (*p* < 0.001, Pyr: *z* = 47.50; PV-In: *z* = 34.57; SOM-In: *z* = 20.85).

Both, spindle density and power in the 7- to 15-Hz spindle frequency band significantly increased from the first to the last third of all SWS episodes (*p* < 0.001; df = 254; *t* = −5.45; *t* = −7.27, respectively; Fig. 3*D*). The increase in spindle band power during SWS correlated with both the parallel increase in calcium activity of spindle-active Pyr cells (*r* = 0.48; *p* < 0.001) and with the decrease in calcium activity of spindle-inactive Pyr cells (*r* = −0.26; *p* < 0.001; Fig. 3*E*).

### Pyr cells active during SOs show stable calcium activity during SWS

**Figure 4.**
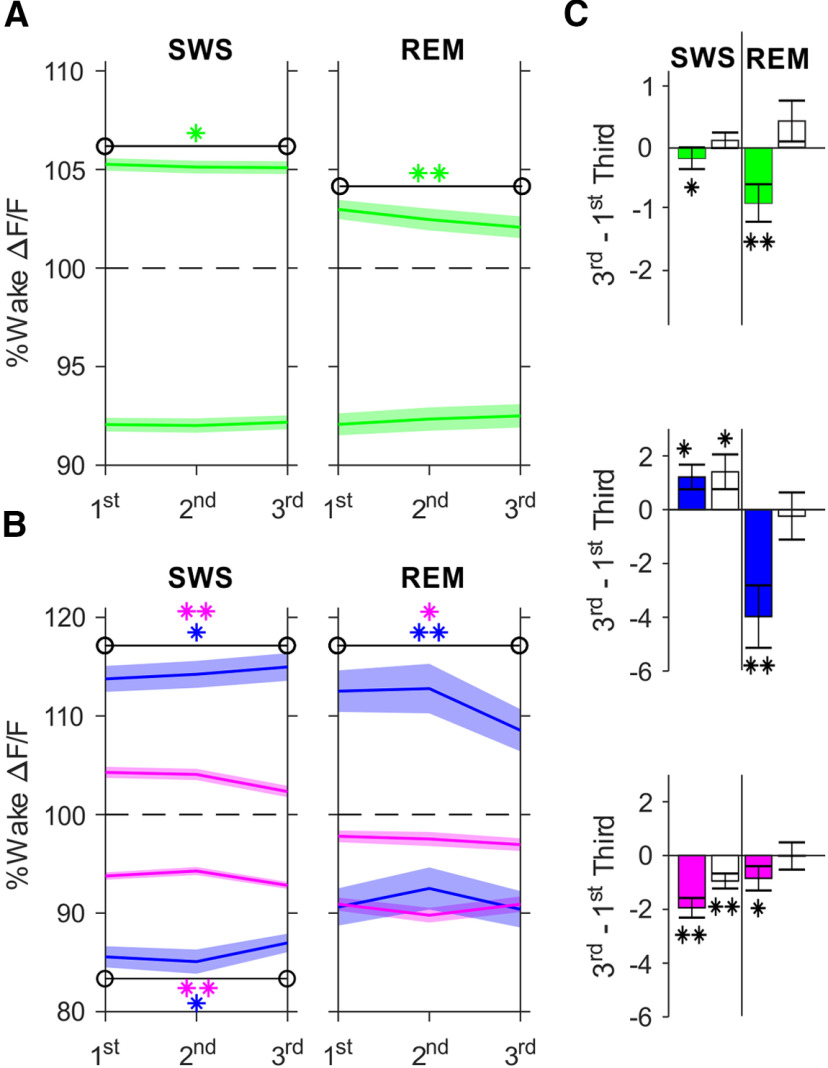
Pyr cells active during SO upstates show stable calcium activity during SWS. ***A***, Mean ± SEM wake-normalized ΔF/F calcium signals of SO-active (upper traces) and SO-inactive (lower traces) Pyr cells (green) during thirds of SWS (*n*: Pyr 255, PV-In 132, SOM-In 123) and REM sleep episodes (*n*: Pyr 145, PV-In 71, SOM-In 74). Significant changes from 1st to last third of SWS and REM episodes are indicated by asterisks (**p < 0.01, *p < 0.05). ***B***, As in ***A***, but for SO-active PV-In (upper lines, blue) and SOM-In (pink) and SO-inactive PV-In and SOM-In (lower blue and pink lines, respectively). ***C***, Mean difference (±SEM) in calcium activity of SO-active (filled bars) and SO-inactive (empty bars) Pyr, PV-In, and SOM-In from 1st to last third (3rd–1st third) of SWS (left) and REM sleep (right) episodes (**p < 0.01, *p < 0.05).

Like spindles, SOs have also been implicated in memory formation (Ngo et al., 2013). However, SOs are also thought to mediate processes of synaptic down-scaling (Tononi and Cirelli, 2014, 2020; González-Rueda et al., 2018). Often, spindles nest into the excitable upstate of the SO. Therefore, to dissociate regulatory functions of SO from those of spindles, here we concentrated on SOs that did not nest spindles. Analog to our approach to spindles, we clustered cells based on their calcium activity during all SO upstates identified in an SWS episode, subtracted by their calcium activity during the remaining time of the SWS episode. The top 20% cells were considered SO-active cells whereas the lowest 20% were considered SO-inactive cells. SO-active Pyr, but not SO-inactive Pyr, cells showed a slight but significant decrease in calcium activity within SWS episodes (*p* < 0.05, *z* = −2.38; Fig. 4). As to the interneuron populations, both SO-active and inactive PV-In increased calcium activity during SWS episodes (*p* < 0.05; active: *z* = 2.38; inactive: *z* = 2.20), while SO-active and inactive SOM-In decreased calcium activity during SWS episodes (*p* < 0.001; active: *z* = −6.00; inactive: *z* = −4.26). Over the course of REM episodes, SO-active Pyr and PV-In decreased calcium activity (Pyr: *p* < 0.001, *z* = −3.58; *F* = 8.72, *p* < 0.05, for 1st/3rd × active/inactive ANOVA interaction, PV-In: *p* < 0.001, *z* = −3.44, *F* = 6.61, *p* < 0.05, for 1st/3rd × active/inactive interaction), whereas SO-active and inactive SOM-In did not express REM-related modulations.

The density of SOs (not nesting spindles) decreased during SWS episodes (*p* < 0.001; *t* = 6.47, df = 254), and there was also a parallel decrease in SWA (power in the 0.1- to 4-Hz band) from the first to the last third of SWS episodes (*p* < 0.001; *t* = 3.97; df = 254). These decreases were not significantly correlated with the dynamics of any of SO-active and SO-inactive cell populations during SWS.

### REM sleep renormalizes calcium activity of spindle-active cells

We found that during REM sleep episodes, spindle-active Pyr as well as the general population of Pyr cells decreased their calcium activity. However, REM sleep also produced an enhanced variance in calcium activity of the general Pyr cell population (Fig. 1), which led us to suspect that the population of spindle-active Pyr might include a subset of cells that is most strongly implicated in memory formation and would, hence, be spared from downregulation during REM sleep (Niethard and Born, 2019). Overall, our analyses did not support this hypothesis. First, variability of calcium activity among spindle-active Pyr did not change from the first to the last third of REM episodes (*p* > 0.1) consistent with homogenous temporal dynamics in this cell population. Second, comparing the 20% of spindle-active Pyr cells showing the strongest upregulation of calcium activity during the prior SWS episode with those 20% showing the weakest upregulation, did not reveal a differential downregulation of calcium activity between these cell subsets during subsequent REM sleep (*p* > 0.4, for 1st/3rd × active/inactive interaction). Finally, assuming that enhanced involvement in spindle-associated memory processing increases the cell's likelihood to escape downregulation during subsequent REM sleep, we tested whether increases in spindle-active Pyr during SWS were followed by diminished decreases or even increases in calcium activity during subsequent REM episodes. However, the respective correlation for spindle-active Pyr was rather low and non-significant (*r* = 0.04, *p* > 0.08).

**Figure 5.**
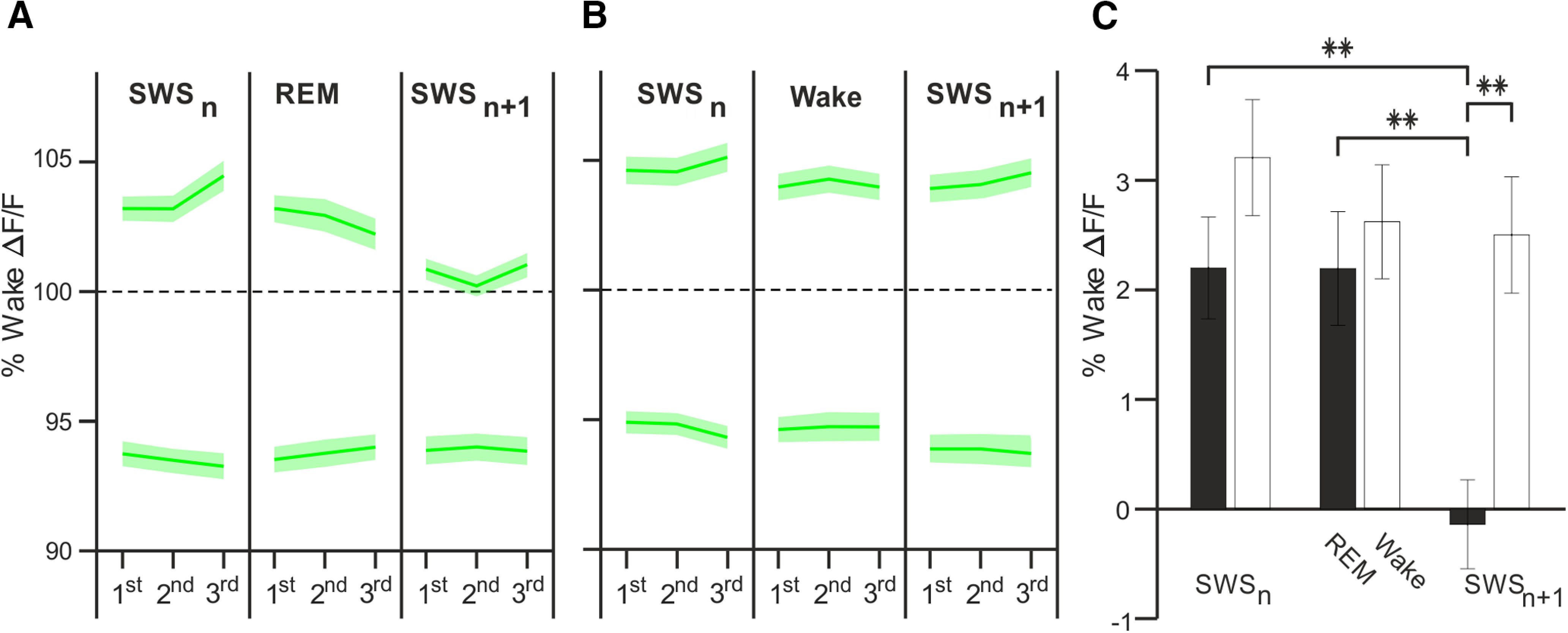
REM sleep downregulates calcium activity of spindle-active Pyr cells. Mean ± SEM wake-normalized ΔF/F calcium signals of spindle-active (upper traces) and spindle-inactive (lower traces) Pyr cells (***A***) during a sequences of sleep episodes comprising a SWS episode (SWS_n_) followed by a REM sleep episode and another SWS episode (SWS_n+1_) and (***B***) during a control sequences where the two SWS episodes, SWS_n_ and SWS_n+1_, are interrupted by a single wake episode. Calcium activity is indicated for thirds of the episodes. ***C***, Mean ± SEM calcium activity in episode sequences containing two succeeding SWS episodes (SWS_n_ and SWS_n+1_) episodes that are either interrupted by a REM sleep episode (black bars) or a wake episode (empty bars). Calcium activity levels for the 1st third of the respective episodes are indicated (normalized by subtracting the level during the prior wake episode, **p < 0.01, *p < 0.05, for pairwise comparisons between conditions). The mean duration of REM and wake episodes, respectively, between SWS_n_ and SWS_n+1_ episodes was 100.95 ± 7.74 and 331.48 ± 39.22 s. Note, REM sleep intervening between the SWS episodes induces a persisting downregulation of Pyr cell calcium activity continuing into the next SWS episode (SWS_n+1_). For analysis of extended periods of consolidated sleep that covered five consecutive SWS episodes (SWS_n_ and SWS_n+5_), see Extended Data Figure 5-1.

We finally examined whether the downregulation of calcium activity in spindle-active Pyr during REM sleep episodes represents a persistent change that is carried over to the next SWS episode. To this end, we compared ΔF/F calcium signals of spindle-active and inactive cells between two consecutive SWS episodes (SWS_n_ vs SWS_n+1_) which were interrupted by a REM sleep episode. A comparison of calcium activity during the 1st thirds of respective SWS episodes revealed that spindle-active Pyr cells indeed exhibited a strong decrease in calcium activity between these consecutive SWS episodes (*p* < 0.001, *z* = 3.98; Fig. 5). No such decrease was observed for spindle-inactive cells (*p* > 0.34, *F* = 17.10, *p* < 0.001, for SWS-1/SWS-2 × active/inactive interaction), or in a further control comparison with calcium activity during consecutive SWS episodes interrupted by a single wake rather than REM sleep episode (*p* > 0.08, *F* = 6.71, *p* < 0.01, for SWS-1/SWS-2 × REM/wake ANOVA interaction). Notably, with a REM sleep episode intervening between the SWS episodes, the ΔF/F signals from spindle-active cells during the second SWS episode fell on average even below the initial level of calcium activity during the preceding wake episode (Fig. 5*C*). Exploratory analyses over extended periods of consolidated sleep covering at least five consecutive SWS episodes confirmed that calcium activity of spindle-active Pyr remained downregulated at the late, i.e., fifth SWS episode, when the first SWS episode of the sleep period was followed by REM sleep (Extended Data Fig. 5-1). It fact, in the beginning of this fifth SWS episode, levels of calcium activity in spindle active cells were very comparable with those of spindle inactive cells. PV-In spindle-active and inactive cell clusters showed no difference between two consecutive SWS episodes (*p* > 0.07 for all analyses). Altogether, these data indicate spindle-active Pyr cells that show unique upregulation of calcium activity during SWS episodes, undergo robust downregulation of calcium activity during subsequent REM sleep.

10.1523/JNEUROSCI.1957-20.2021.f5-1 Extended Data Figure 5-1 REM sleep persistently downregulates activity of spindle-active Pyr cells. Mean ± SEM wake-normalized ΔF/F calcium signals of spindle-active and spindle-inactive Pyr cells in an analysis of extended periods of consolidated sleep that covered at least five consecutive SWS-episodes (*n* = 86, mean ± SEM period duration 55.48 ± 9.16 min). Spindle-active and inactive cells were defined by the cell's activity (25% most active and least active Pyr, respectively) in the first SWS_n_ episode of the sleep periods, and only sleep periods were included where SWS_n_ was followed by REM sleep. ***A***, Mean ± SEM calcium activity during the first (SWS_n_) and fifth (SWS_n+5_) episode (1st third of episode) for spindle active (black) and inactive Pyr cells (empty bars). ***B***, Mean ± SEM time courses of wake-normalized ΔF/F calcium signals for the same spindle-active (upper traces) and spindle-inactive Pyr cells (lower traces) over the thirds (1st, 2nd, 3rd) of the first SWS episode (SWS_n_), the succeeding REM episode, and the fifth SWS episode (SWS_n+5_) of the sleep period; **p < 0.001, for comparisons between groups or pairwise comparisons between SWS_n_ and SWS_n+5_. Note, REM sleep following the initial SWS episode induces a downregulation of calcium activity in spindle-active Pyr cell that persists into the fifth SWS episode (SWS_n+5_) such that calcium activity levels between spindle-active and spindle-inactive Pyr cell populations are quite comparable at this late SWS episode. The effect cannot be ascribed to fluorescence bleaching occurring across extended recording intervals which would have affected calcium activity of spindle active and inactive cells in the same direction. The result also fits well with previous findings indicating a narrowing of spike activity distributions across sleep (Watson et al., 2016; Clawson et al., 2018). Download Figure 5-1, TIF file.

## Discussion

**Figure 6.**
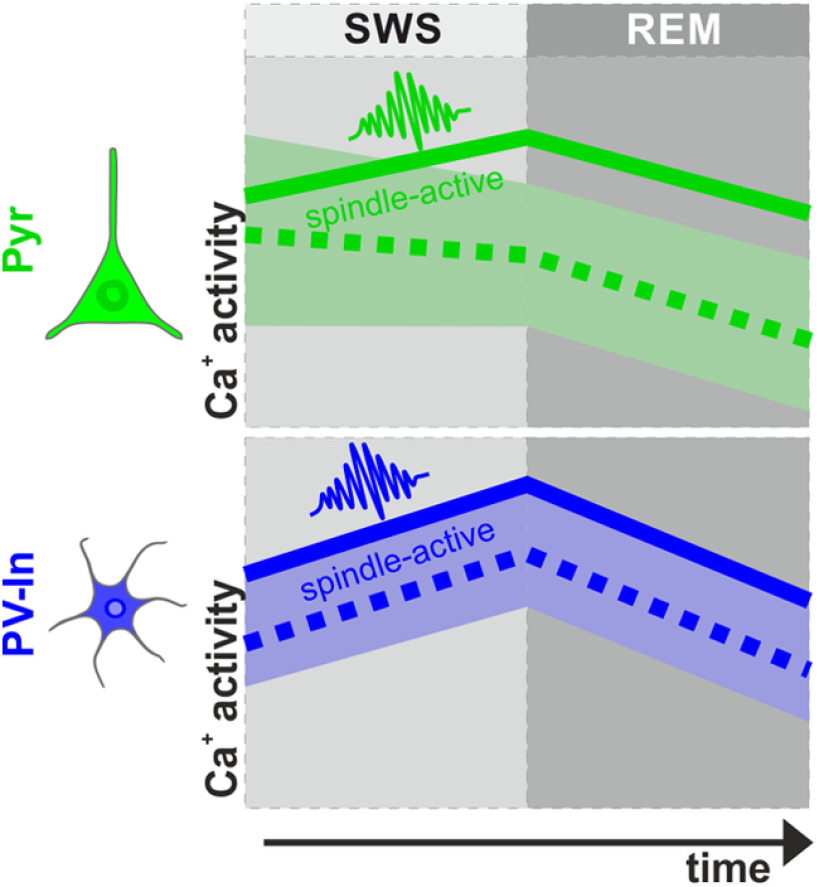
Summary of the studies' main findings. During SWS spindle-active Pyr cells increase activity (dark green line) while the remaining Pyr cells decrease their activity (light green shaded area, dashed line shows mean of overall Pyr cell population). Simultaneously during SWS spindle-active PV-In cells (dark blue line) as well as the rest of the PV-In cell population (light blue shaded area, dashed line shows mean of overall PV-In population) both increase their activity. Subsequent REM sleep is accompanied by reduced activity of all cells, indicating a general network downregulation that is dependent on REM theta energy. Therefore, the downregulation of Pyr cells during SWS potentially derives from increased inhibition via PV-In, whereas during REM sleep, PV-In cells also reduce their activity, and thus, their inhibitory input cannot explain the general down regulation here. Note, we arbitrarily defined spindle-active cells by the 20% of the respective total cell population showing highest activity during spindles, with this approach precluding the specification of exact numbers of spindle-active cells. Moreover, the schema does not integrate our findings regarding the dynamics of calcium activity during longer wake episodes that followed a SWS episode and thereby interrupted the ongoing sleep process.

We used *in vivo* calcium imaging of cortical circuits in naturally sleeping mice to dissociate neuronal populations whose calcium activity undergo downregulation across sleep from those which are either not downregulated or even upregulated. We found that during both episodes of SWS and REM sleep, the variability in calcium activity levels increased in the population of excitatory Pyr cells representing the great majority of cortical neurons. This finding supports the view that sleep does not uniformly regulate calcium activity in this cell population. On average, the increase in variability of calcium activity was accompanied by a decrease in Pyr cell calcium activity over the course of both SWS and REM sleep episodes, confirming that sleep globally downregulates cortical network activity. However, in opposition to the average trend, we also identified Pyr cells that were characterized by high calcium activity during sleep spindles, and whose calcium activity distinctly increased over the course of SWS episodes. Because spindles are known to promote synaptic plasticity underlying memory formation (Steriade and Timofeev, 2003; Rosanova and Ulrich, 2005; Seibt et al., 2017; Niethard et al., 2018; Puentes-Mestril et al., 2019; Fernandez and Lüthi, 2020), this finding concurs with the notion that sleep up-scales subpopulations of Pyr cells involved in memory formation. Surprisingly, the same subpopulation of spindle-active Pyr cells underwent profound downregulation of calcium activity during subsequent REM sleep episodes (for a summary of results, see Fig. 6).

Our finding that both SWS and REM sleep episodes produce a robust increase in the variability of calcium activity levels in the Pyr cell population contrasts with electrophysiological findings that demonstrate a shrinking rather than widening of firing rate distributions across sleep (Watson et al., 2016; Clawson et al., 2018). A more recent study (Miyawaki et al., 2019) carefully accounting for regression to the mean of firing rates, showed a widening of firing rate distributions across REM sleep, which is consistent with the present findings, but still a shrinking of firing rate distributions across SWS episodes. Specifically, all these studies found that neurons with high firing rates show a strong decrease in spiking activity over the course of SWS, while neurons with low firing rates slightly increase spiking activity. Indeed, this more nuanced understanding was confirmed in the present study when the wake episode preceding the respective episodes of SWS was considered: Pyr cells showing high calcium activity during this wake episode showed a pronounced decrease in calcium activity during the subsequent SWS episodes whereas the wake-inactive cells tended to increase in calcium activity (Extended Data Fig. 3-1). Notably, correlational analyses also showed that Pyr cells whose calcium activity was upregulated over the course of this wake episode were more likely to have upregulated calcium activity during the succeeding SWS episode. In combination, these results can explain the increase in variance of Pyr cell calcium activity levels over the course of SWS, and are in line with the notion of a twofold function of this sleep stage, i.e., on the one hand to homeostatically downregulates activity in a manner that captures cells that were particularly highly active during prior wakefulness and, on the other hand, to protect or even upregulate the activity of Pyr cells that showed dynamic upregulation during prior wakefulness and whose activity might therefore be linked to information encoding.

However, when comparing the present findings based on calcium imaging of large cell populations with previous electrophysiological findings, further factors need to be considered as well. Imaging of calcium activity also measures neurons with very low spiking activity, which are typically missed in electrophysiological recordings (Brecht et al., 2003). Importantly, rather than extracting calcium spikes, we decided to base our analyses on the original fluorescence signal, thus allowing the inclusion of subthreshold calcium activity in neurons which are likely implicated in sleep-dependent downregulation processes (Bartram et al., 2017). This approach is commonly used for studying brain state-dependent changes of calcium activity that develop at a slow pace over several minutes in distinct cortical cell populations (Cox et al., 2016; Oesch et al., 2020). Extracting calcium spikes would not be an optimal approach in these conditions, as the relatively low temporal resolution of the signal leads to distorted spike detection, especially in the presence of burst-like spike activity which typically accompanies spindles (Peyrache et al., 2011; Fernandez and Lüthi, 2020). Another aspect relevant for the comparison of our findings with electrophysiological studies is that cortical activity dynamics can be layer-specific. Whereas here we focus on the calcium activity of layer 2/3 cells, spiking activity has mainly been studied in layer 5 neurons. Finally, our measurement of calcium activity in the cell soma is not an immediate reflection of the neuron's (electrical) spiking activity in terms of action potentials, but rather reflects the integration of spatially extended activity over time with a lower temporal resolution. While our approach provides insights into slow changes of cortical calcium activity during sleep, we were not able to investigate calcium activity or spikes at defined phases of spindle or theta oscillations as this would require distinctly higher sampling rates and a transformed binary signal that allows the unbiased coding of “spikes” occurring in rapid succession (i.e., during bursts).

The mechanisms underlying the average decrease in Pyr cell calcium activity during SWS and REM episodes are unclear but might differ between these sleep stages. For SWS, previous work has linked SWA to down-scaling of synaptic networks (Gulati et al., 2017; González-Rueda et al., 2018; Tononi and Cirelli, 2020). However, in the present study, no consistent correlations were found between measures of SWA and changes in Pyr cell calcium activity over SWS episodes. Rather than a consequence of weakened and decreased synaptic connections, the decrease in calcium activity of excitatory Pyr cells appeared to be a consequence of increased inhibitory inputs to these cells, as PV-In calcium activity distinctly increased over the course of SWS episodes. The increase in calcium activity of PV-In, which effectively inhibit the soma of Pyr cells, could itself account for the downregulation of Pyr cell calcium activity over the course of SWS, negating the need for synaptic re-normalization. On the other side, the decrease in Pyr cell calcium activity during REM sleep episodes, unlike that during SWS, was not accompanied by any increase in calcium activity of the major inhibitory neurons. In fact, calcium activity of PV-In even significantly decreased from the first to the last third of REM episodes, while SOM-In calcium activity remained unchanged. Notably, the decrease in Pyr calcium activity persisted into subsequent SWS episodes. A supplementary analysis on extended periods of consolidated sleep (covering five consecutive SWS episodes) confirmed that Pyr cells identified as spindle-active cells in the initial SWS episode of the sleep period continued to show downregulated low levels of calcium activity even at the late, i.e., fifth SWS episode of this sleep period. In combination, this pattern is consistent with the view that decreasing levels of Pyr calcium activity during REM sleep are a consequence of synaptic re-normalization, rather than of sleep stage-specific inhibition. However, given that we assessed calcium activity, any conclusion with respect to synaptic changes remains tentative. The REM-sleep related decrease in Pyr cell calcium activity was also positively correlated with EEG theta energy (i.e., theta power integrated over time). Similar evidence suggesting a contribution of theta activity to downregulating network activity has been derived from correlational analyses of hippocampal spike activity across triplets of SWS-REM-SWS episodes (Grosmark et al., 2012). However, evidence for the involvement of theta activity in synaptic down-scaling processes is otherwise scarce and indirect (Poe et al., 2000). Further studies that manipulate REM sleep while directly measuring synaptic strength are needed to establish a causal role of REM sleep in synaptic re-normalization processes.

We identified a subpopulation of Pyr neurons that showed maximal calcium activity during spindles, and which, opposite to the global deactivation across SWS, displayed a substantial increase in calcium activity during SWS. This is a central finding of our study and it supports the idea that during sleep, and SWS in particular, an upregulation occurs in cell assemblies which were specifically involved in prior encoding of information, while network activity is globally downregulated. The upregulation of calcium activity during SWS was specific to spindle-active Pyr and PV-In. It was not seen in wake-active cells or cells most active during the upstate of SOs, both of which exhibited downregulated calcium activity over the course of SWS. In the latter analyses, only SOs that did not nest a spindle were considered, which allowed clear discrimination between the function of spindles compared with that of SOs. Spindles have been shown to promote the formation of memory and underlying synaptic plastic processes, like long-term potentiation (Rosanova and Ulrich, 2005). At the level of cortical microcircuits, they are characterized by increased activity of Pyr cells in the presence of distinctly enhanced somatic inhibition of these cells via PV-In, i.e., a constellation facilitating plastic synaptic changes in response to afferent inputs to the distal dendrites of these cells (Seibt et al., 2017; Niethard et al., 2018). Fittingly, the present study reveals a parallel increase in the calcium activity of spindle-active PV-In over the course of SWS. Moreover, the increase in Pyr cell calcium activity was paralleled by an increase in spindle density across SWS, a phenomenon well-known from previous studies (Dijk et al., 1993). Spindles generated in thalamic networks with thalamo-cortical projections to both PV-In and Pyr (Porter et al., 2001; Schubert et al., 2003) could represent the driving force responsible for upregulating the activity of these circuits during memory processing (Jiang et al., 2017).

Unexpectedly, during REM sleep calcium activity of spindle-active Pyr cells showed dynamics that were very similar to the overall Pyr cell population, i.e., calcium activity of these cells was uniformly downregulated and this downregulation persistently extended into subsequent SWS episodes. In parallel, spindle-active PV-In neurons were also downregulated during REM sleep episodes, while calcium activity levels of spindle-active SOM-In remained unchanged. This pattern is consistent with synaptic re-scaling processes which mediate the persistent downregulation of Pyr cell activity during REM sleep, and concurs with evidence from structural imaging of mice layer 5 Pyr cells which demonstrated that REM sleep prunes postsynaptic dendritic spines that were newly formed during prior motor learning in these neurons (Li et al., 2017). In light of the present data, the Pyr cells with newly formed synapses following motor learning would be expected to constitute the population of spindle-active cells. However, that study also showed that REM sleep simultaneously maintains and strengthens a fraction of the newly formed synapses, which was critical for motor improvement seen after sleep. Diverging from this finding, we were unable to identify a subgroup of spindle-active cells that did not undergo downregulation during REM sleep. There are two feasible explanations. First, it is possible that REM sleep-associated strengthening of synapses might only occur in neurons with synapses that are simultaneously eliminated. In our method, measurement was focused on the soma of Pyr cells, and therefore, data represent the integrated calcium activity of the entire neuron. Synaptic elimination may therefore mask synaptic strengthening in our data. Second, in our study the mice were not trained on a specific learning task before sleep (though it should be considered that sleeping in head-fixed conditions could be aversive and thereby prompt the mouse to encode information whenever it is awake). While this on the one hand could represent a study limitation, on the other hand, it is specifically this lack of learning that may have led to a uniform down-scaling of all newly formed synapses. The latter option suggests the existence of another mechanism beyond spindles that influences, e.g., based on the novelty of the encoded information, whether a newly formed representation is maintained or eliminated during REM sleep (Poe et al., 2000). This is a promising hypothesis that should be tested in future research.

## References

[B1] Bartram J, Kahn MC, Tuohy S, Paulsen O, Wilson T, Mann EO (2017) Cortical Up states induce the selective weakening of subthreshold synaptic inputs. Nat Commun 8:665. 10.1038/s41467-017-00748-5 28939859 PMC5610171

[B2] Boyce R, Glasgow SD, Williams S, Adamantidis A (2016) Causal evidence for the role of REM sleep theta rhythm in contextual memory consolidation. Science 352:812–816. 10.1126/science.aad5252 27174984

[B3] Brecht M, Roth A, Sakmann B (2003) Dynamic receptive fields of reconstructed pyramidal cells in layers 3 and 2 of rat somatosensory barrel cortex. J Physiol 553:243–265. 10.1113/jphysiol.2003.044222 12949232 PMC2343497

[B4] Buzsáki G (2002) Theta oscillations in the hippocampus. Neuron 33:325–340. 10.1016/s0896-6273(02)00586-x 11832222

[B5] Chauvette S, Seigneur J, Timofeev I (2012) Sleep oscillations in the thalamocortical system induce long-term neuronal plasticity. Neuron 75:1105–1113. 10.1016/j.neuron.2012.08.034 22998877 PMC3458311

[B6] Clawson BC, Durkin J, Suresh AK, Pickup EJ, Broussard CG, Aton SJ (2018) Sleep promotes, and sleep loss inhibits, selective changes in firing rate, response properties and functional connectivity of primary visual cortex neurons. Front Syst Neurosci 12:40. 10.3389/fnsys.2018.00040 30245617 PMC6137342

[B7] Cox J, Pinto L, Dan Y (2016) Calcium imaging of sleep-wake related neuronal activity in the dorsal pons. Nat Commun 7:10763–10767. 10.1038/ncomms10763 26911837 PMC4773416

[B8] David F, Schmiedt JT, Taylor HL, Orban G, Di Giovanni G, Uebele VN, Renger JJ, Lambert RC, Leresche N, Crunelli V (2013) Essential thalamic contribution to slow waves of natural sleep. J Neurosci 33:19599–19610. 10.1523/JNEUROSCI.3169-13.2013 24336724 PMC3858629

[B9] de Vivo L, Bellesi M, Marshall W, Bushong EA, Ellisman MH, Tononi G, Cirelli C (2017) Ultrastructural evidence for synaptic scaling across the wake/sleep cycle. Science 355:507–510. 10.1126/science.aah5982 28154076 PMC5313037

[B10] Diering GH, Nirujogi RS, Roth RH, Worley PF, Pandey A, Huganir RL (2017) Homer1a drives homeostatic scaling-down of excitatory synapses during sleep. Science 355:511–515. 10.1126/science.aai8355 28154077 PMC5382711

[B11] Dijk DJ, Hayes B, Czeisler CA (1993) Dynamics of electroencephalographic sleep spindles and slow wave activity in men: effect of sleep deprivation. Brain Res 626:190–199. 10.1016/0006-8993(93)90579-c 8281430

[B12] Dombeck DA, Khabbaz AN, Collman F, Adelman TL, Tank DW (2007) Imaging large-scale neural activity with cellular resolution in awake, mobile mice. Neuron 56:43–57. 10.1016/j.neuron.2007.08.003 17920014 PMC2268027

[B13] Fernandez LMJ, Lüthi A (2020) Sleep spindles: mechanisms and functions. Physiol Rev 100:805–868. 10.1152/physrev.00042.2018 31804897

[B14] González-Rueda A, Pedrosa V, Feord RC, Clopath C, Paulsen O (2018) Activity-dependent downscaling of subthreshold synaptic inputs during slow-wave-sleep-like activity in vivo. Neuron 97:1244–1252.e5. 10.1016/j.neuron.2018.01.047 29503184 PMC5873548

[B15] Grosmark AD, Mizuseki K, Pastalkova E, Diba K, Buzsáki G (2012) REM sleep reorganizes hippocampal excitability. Neuron 75:1001–1007. 10.1016/j.neuron.2012.08.015 22998869 PMC3608095

[B16] Gulati T, Guo L, Ramanathan DS, Bodepudi A, Ganguly K (2017) Neural reactivations during sleep determine network credit assignment. Nat Neurosci 20:1277–1284. 10.1038/nn.4601 28692062 PMC5808917

[B17] Huber R, Ghilardi MF, Massimini M, Tononi G (2004) Local sleep and learning. Nature 430:78–81. 10.1038/nature02663 15184907

[B18] Jiang X, Shen S, Cadwell CR, Berens P, Sinz F, Ecker a. S, Patel S, Tolias a. S (2015) Principles of connectivity among morphologically defined cell types in adult neocortex. Science 350:aac9462. 10.1126/science.aac9462 26612957 PMC4809866

[B19] Jiang X, Shamie IK, Doyle W, Friedman D, Dugan P, Devinsky O, Eskandar E, Cash SS, Thesen T, Halgren E (2017) Replay of large-scale spatio-temporal patterns from waking during subsequent NREM sleep in human cortex. Sci Rep 7:17380. 10.1038/s41598-017-17469-w 29234075 PMC5727134

[B20] Kim J, Gulati T, Ganguly K (2019) Competing roles of slow oscillations and delta waves in memory consolidation versus forgetting. Cell 179:514–526.e13. 10.1016/j.cell.2019.08.040 31585085 PMC6779327

[B21] Klinzing JG, Niethard N, Born J (2019) Mechanisms of systems memory consolidation during sleep. Nat Neurosci 22:1598–1610. 10.1038/s41593-019-0467-3 31451802

[B22] Latchoumane CFV, Ngo HVV, Born J, Shin HS (2017) Thalamic spindles promote memory formation during sleep through triple phase-locking of cortical, thalamic, and hippocampal rhythms. Neuron 95:424–435.e6. 10.1016/j.neuron.2017.06.025 28689981

[B23] Li W, Ma L, Yang G, Gan WB (2017) REM sleep selectively prunes and maintains new synapses in development and learning. Nat Neurosci 20:427–416. 10.1038/nn.4479 28092659 PMC5535798

[B24] Markram H, Toledo-Rodriguez M, Wang Y, Gupta A, Silberberg G, Wu C (2004) Interneurons of the neocortical inhibitory system. Nat Rev Neurosci 5:793–807. 10.1038/nrn1519 15378039

[B25] Miyawaki H, Watson BO, Diba K (2019) Neuronal firing rates diverge during REM and homogenize during non-REM. Sci Rep 9:689. 10.1038/s41598-018-36710-8 30679509 PMC6345798

[B26] Ngo HVV, Martinetz T, Born J, Mölle M (2013) Auditory closed-loop stimulation of the sleep slow oscillation enhances memory. Neuron 78:545–553. 10.1016/j.neuron.2013.03.006 23583623

[B27] Niethard N, Born J (2019) Back to baseline: sleep recalibrates synapses. Nat Neurosci 22:149–151. 10.1038/s41593-018-0327-6 30617259

[B28] Niethard N, Hasegawa M, Itokazu T, Oyanedel CN, Born J, Sato TR (2016) Sleep-stage-specific regulation of cortical excitation and inhibition. Curr Biol 26:2739–2749. 10.1016/j.cub.2016.08.035 27693142

[B29] Niethard N, Burgalossi A, Born J (2017) Plasticity during sleep is linked to specific regulation of cortical circuit activity. Front Neural Circuits 11:1–9. 28966578 10.3389/fncir.2017.00065 PMC5605564

[B30] Niethard N, Ngo H-VV, Ehrlich I, Born J (2018) Cortical circuit activity underlying sleep slow oscillations and spindles. Proc Natl Acad Sci USA 115:E9220–E9229. 10.1073/pnas.1805517115 30209214 PMC6166829

[B31] Oesch LT, Gazea M, Gent TC, Bandarabadi M, Herrera CG, Adamantidis AR (2020) REM sleep stabilizes hypothalamic representation of feeding behavior. Proc Natl Acad Sci USA 117:19590–19598. 10.1073/pnas.1921909117 32732431 PMC7430996

[B32] Oyanedel CN, Durán E, Niethard N, Inostroza M, Born J (2020) Temporal associations between sleep slow oscillations, spindles and ripples. Eur J Neurosci 52:4762–4778. 10.1111/ejn.14906 32654249

[B33] Peyrache A, Battaglia FP, Destexhe A (2011) Inhibition recruitment in prefrontal cortex during sleep spindles and gating of hippocampal inputs. Proc Natl Acad Sci USA 108:17207–17212. 10.1073/pnas.1103612108 21949372 PMC3193185

[B34] Poe GR, Nitz DA, McNaughton BL, Barnes CA (2000) Experience-dependent phase-reversal of hippocampal neuron firing during REM sleep. Brain Res 855:176–180. 10.1016/s0006-8993(99)02310-0 10650147

[B35] Pologruto TA, Sabatini BL, Svoboda K (2003) ScanImage: flexible software for operating laser scanning microscopes. Biomed Eng Online 2:13. 10.1186/1475-925X-2-13 12801419 PMC161784

[B36] Porter JT, Johnson CK, Agmon A (2001) Diverse types of interneurons generate thalamus-evoked feedforward inhibition in the mouse barrel cortex. J Neurosci 21:2699–2710. 10.1523/JNEUROSCI.21-08-02699.2001 11306623 PMC6762510

[B37] Puentes-Mestril C, Roach J, Niethard N, Zochowski M, Aton SJ (2019) How rhythms of the sleeping brain tune memory and synaptic plasticity. Sleep 42:zsz095. 10.1093/sleep/zsz095 31100149 PMC6612670

[B38] Rasch B, Born J (2013) About sleep's role in memory. Physiol Rev 93:681–766. 10.1152/physrev.00032.2012 23589831 PMC3768102

[B39] Rosanova M, Ulrich D (2005) Pattern-specific associative long-term potentiation induced by a sleep spindle-related spike train. J Neurosci 25:9398–9405. 10.1523/JNEUROSCI.2149-05.2005 16221848 PMC6725710

[B40] Sawangjit A, Oyanedel CN, Niethard N, Salazar C, Born J, Inostroza M (2018) The hippocampus is crucial for forming non-hippocampal long-term memory during sleep. Nature 564:109–113. 10.1038/s41586-018-0716-8 30429612

[B41] Schubert D, Kötter R, Zilles K, Luhmann HJ, Staiger JF (2003) Cell type-specific circuits of cortical layer IV spiny neurons. J Neurosci 23:2961–2970. 12684483 10.1523/JNEUROSCI.23-07-02961.2003 PMC6742105

[B42] Seibt J, Richard CJ, Sigl-Glöckner J, Takahashi N, Kaplan DI, Doron G, de Limoges D, Bocklisch C, Larkum ME (2017) Cortical dendritic activity correlates with spindle-rich oscillations during sleep in rodents. Nat Commun 8:684. 10.1038/s41467-017-00735-w 28947770 PMC5612962

[B43] Spano GM, Banningh SW, Marshall W, L de V, Bellesi M, Loschky SS, Tononi G, Cirelli C (2019) Sleep deprivation by exposure to novel objects increases synapse density and axon-spine interface in the hippocampal CA1 region of adolescent mice. J Neurosci 39:6613–6625. 31263066 10.1523/JNEUROSCI.0380-19.2019 PMC6703893

[B44] Steriade M, Timofeev I (2003) Neuronal plasticity in thalamocortical networks during sleep and waking oscillations. Neuron 37:563–576. 10.1016/s0896-6273(03)00065-5 12597855

[B45] Tononi G, Cirelli C (2014) Sleep and the price of plasticity: from synaptic and cellular homeostasis to memory consolidation and integration. Neuron 81:12–34. 10.1016/j.neuron.2013.12.025 24411729 PMC3921176

[B46] Tononi G, Cirelli C (2020) Sleep and synaptic down-selection. Eur J Neurosci 51:413–421. 10.1111/ejn.14335 30614089 PMC6612535

[B47] Watson BO, Levenstein D, Greene JP, Gelinas JN, Buzsáki G (2016) Network homeostasis and state dynamics of neocortical sleep. Neuron 90:839–852. 10.1016/j.neuron.2016.03.036 27133462 PMC4873379

[B48] Yang G, Lai CSW, Cichon J, Ma L, Li W, Gan W-B (2014) Sleep promotes branch-specific formation of dendritic spines after learning. Science 344:1173–1178. 10.1126/science.1249098 24904169 PMC4447313

