# Supplementary figures and images for: Cell-Type-Specific Dynamics of Calcium Activity in Cortical Circuits over the Course of Slow-Wave Sleep and Rapid Eye Movement Sleep
Source: J Neurosci. 2021 May 12;41(19):4212–22. doi: 10.1523/JNEUROSCI.1957-20.2021 (PMC8143210; doi:10.1523/JNEUROSCI.1957-20.2021)

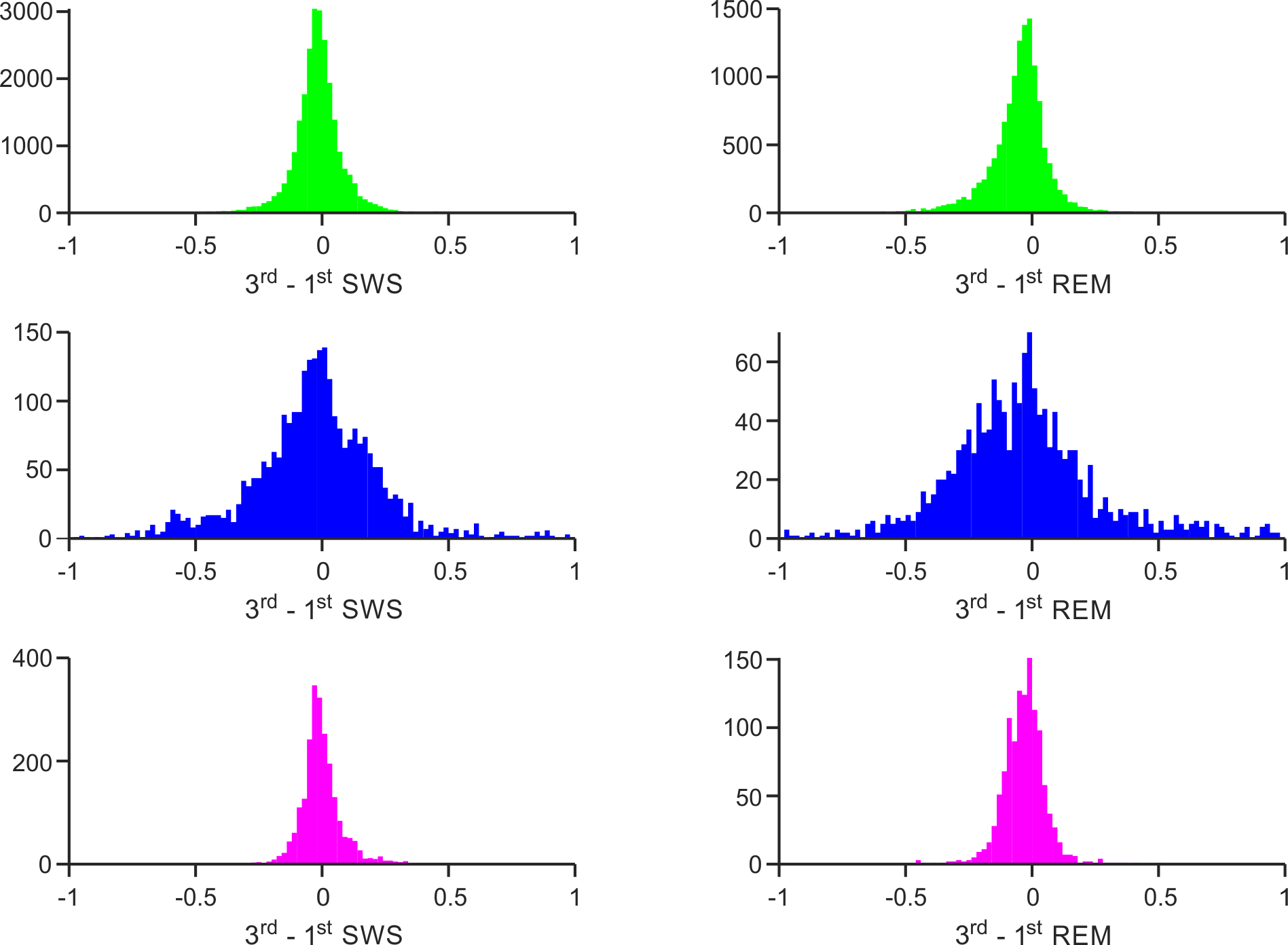

Supplement: Extended Data Figure 1-1 — Distributions of changes in calcium activity during episodes of SWS and REM sleep. Distributions of changes in the wake normalized ΔF/F calcium signals from 1st to last third (3rd–1st third) of SWS (left) and REM sleep (right) episodes for Pyr (green), PV-In (blue), and SOM-In (pink); y-axis indicates number of cells, x-axis change in normalized ΔF/F signal. Download Figure 1-1, TIF file. [file ns-JN-RM-1957-20-s01.tif]

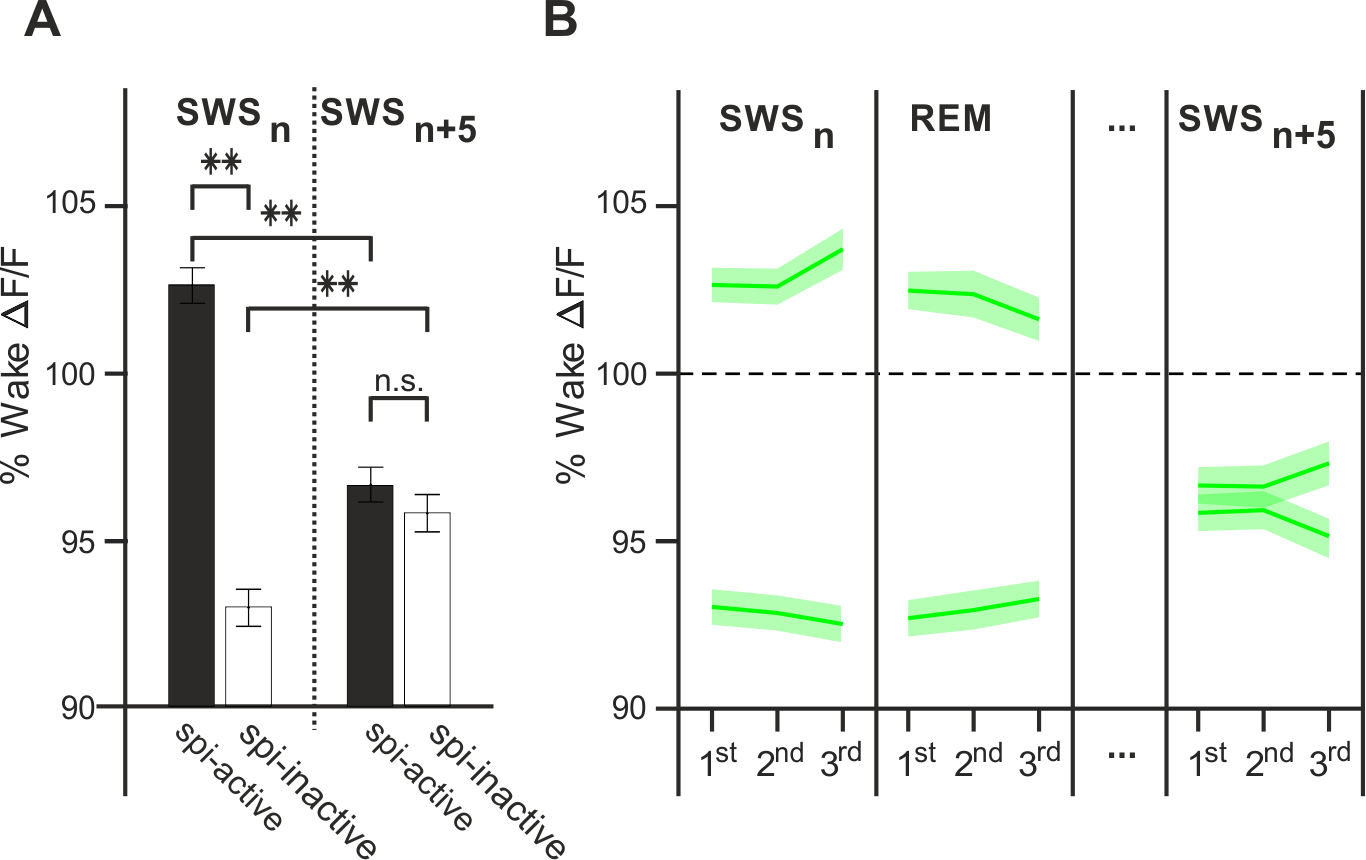

Supplement: Extended Data Figure 3-1 — Pyr cells showing generally high calcium activity during wakefulness reduce their activity during SWS. A, Mean ± SEM wake-normalized ΔF/F calcium signals of wake-active (upper lines) and wake-inactive (lower lines) Pyr cells (green) during thirds of SWS (n: Pyr 255, PV-In 132, SOM-In 123) and REM sleep episodes (n: Pyr 145, PV-In 71, SOM-In 74). Significant changes from 1st to last third of SWS and REM episodes are indicated by asterisks in the respective color (**p < 0.01, *p < 0.05). B, As in A, but for wake-active PV-In (upper lines, blue) and SOM-In (pink) and wake-inactive PV-In and SOM-In (lower blue and pink lines, respectively). C, Mean difference (±SEM) in calcium activity of wake-active (filled bars) and wake-inactive (empty bars) Pyr, PV-In, and SOM-In from 1st to last third (3rd–1st third) of SWS (left) and REM sleep (right) episodes (**p < 0.01, *p < 0.05). “Wake-active” cells refer to the top 20% of the cells (of the respective population) with the highest average calcium activity during the wake episode preceding the respective SWS episode. Correspondingly, “wake-inactive” cells refer to the 20% cells with the lowest calcium activity during this wake episode. Download Figure 3-1, TIF file. [file ns-JN-RM-1957-20-s05.tif]
