# Supplementary material for: Cell-Type-Specific Dynamics of Calcium Activity in Cortical Circuits over the Course of Slow-Wave Sleep and Rapid Eye Movement Sleep
Source: J Neurosci. 2021 May 12;41(19):4212–22. doi: 10.1523/JNEUROSCI.1957-20.2021 (PMC8143210; doi:10.1523/JNEUROSCI.1957-20.2021)
Supplement: Extended Data Figure 1-2 — Mean ± SEM SWS and REM sleep episode duration for PV-cre and SOM-cre animals in seconds. Note, sleep stage episodes <30 s were excluded from analyses. Download Figure 1-2, DOCX file. [file ns-JN-RM-1957-20-s02.docx]

|  | Mean SWS episode duration ± SEM (in s) | Mean REM episode duration ± SEM (in s) |
| --- | --- | --- |
| PV-cre | 150.24 ± 8.49 (n = 132) | 77.74 ± 5.87 (n = 71) |
| SOM-cre | 112.12 ± 4.08 (n = 123) | 85.12 ± 5.7 (n = 74) |

**Figure 1-2.** Mean ± SEM SWS and REM sleep episode duration for PV-cre and SOM-cre animals in seconds. Note, sleep stage episodes <30 s were excluded from analyses.
