# Supplementary material for: Cell-Type-Specific Dynamics of Calcium Activity in Cortical Circuits over the Course of Slow-Wave Sleep and Rapid Eye Movement Sleep
Source: J Neurosci. 2021 May 12;41(19):4212–22. doi: 10.1523/JNEUROSCI.1957-20.2021 (PMC8143210; doi:10.1523/JNEUROSCI.1957-20.2021)
Supplement: Extended Data Figure 1-3 — Total imaging time per session for PV-cre and SOM-cre animals in minutes. The total imaging time was for PV-cre animals 31.75 h, and for SOM-cre animals 40.26 h. Download Figure 1-3, DOCX file. [file ns-JN-RM-1957-20-s03.docx]

| # session | PV-cre | SOM-cre |
| --- | --- | --- |
| 1 | 434.05 | 456.48 |
| 2 | 230.76 | 459.05 |
| 3 | 405.79 | 276.54 |
| 4 | 63.93 | 281.39 |
| 5 | 293.32 | 301.01 |
| 6 | 477.18 | 287.14 |
| 7 |  | 353.80 |

**Figure 1-3.** Total imaging time per session for PV-cre and SOM-cre animals in minutes. The total imaging time was for PV-cre animals 31.75h, and for SOM-cre animals 40.26h.
